# The remnant axial cortical length of the proximal femur in pertrochanteric fractures: a three-dimensional computed tomography study and its clinical implications

**DOI:** 10.1186/s12891-023-07059-5

**Published:** 2023-12-05

**Authors:** Bo Li, Sun-jun Hu, Shi-min Chang, Zhen Wei, Shou-chao Du, Wen-feng Xiong

**Affiliations:** Department of Orthopedic Surgery, Yangpu Hospital, Tongji University School of Medicine, Shanghai, 200090 China

**Keywords:** Pertrochanteric fracture, Circumferential proximal cortex, Remnant anterior cortex, Fracture reduction

## Abstract

**Background:**

Cortical buttress are important factors for postoperative stable reconstruction of per/inter-trochanteric fractures. The study aimed to measure the remnant axial cortical length (RACL) of the proximal circumference of the femur, and to determine which part of the RACL can be used reliably to postoperatively sustain the head–neck fragment as a cortical support pattern.

**Methods:**

Eighty patients with trochanteric hip fractures admitted from January 2015 to January 2016 were included in a retrospective study. Their pre-operative computed tomography (CT) images were used to form 3D-CT reconstructions via Mimics software. After simulated rotation and movement for fracture reduction, the RACL, its three component parts—namely, the remnant anterior cortex (RAC), remnant lateral cortex (RLC), and remnant posterior cortex (RPC) —the γ angle between the anterior and posterior cortex, and the Hsu’s lateral wall thickness (LWT) were evaluated.

**Results:**

Patients with an A1 fracture (21/80) had a longer RACL (88.8 ± 15.8 mm) than those with an A2 fracture (60.0 ± 11.9 mm; *P* < 0.01). The RAC, RLC, and RPC of the RACL in A1 fractures were also significantly longer than those in A2 fractures (*P* < 0.001). However, the most significant difference among the three components of the RACL was in the RPC, which was 27.3 ± 7.8 mm in A1 fractures and 9.2 ± 6.6 mm in A2 fractures. In addition, the coefficient of variation of the RAC was only 20.0%, while that of the RPC was 75.5%. The average γ angle in A1 fractures was 16.2 ± 13.1°, which was significantly smaller than that in A2 fractures, which was 40.3 ± 14.5° (*P* < 0.001). There was a significant statistical difference in the LWT between A1 and A2 fractures (*P* < 0.001). There were significant differences in the RACL, RAC, RLC, RPC, γ angle, and LWT among the five subtypes (*P* < 0.001).

**Conclusions:**

The RAC is relatively stable in pertrochanteric fractures. Fracture reduction through a RAC buttress may help to enhance the postoperative stable reconstruction of per/inter-trochanteric fractures and make possible good mechanical support for fracture healing.

## Introduction

Geriatric hip fractures continue to increase in frequency in the aging population all over the world, and per/inter-trochanteric femur fractures make up a significant proportion of these injuries, and they remain a substantial challenge [1]. Regardless of whether intramedullary or extramedullary fixation systems are used in the internal fixation of trochanteric hip fractures, the implant, usually a lag screw or helical blade, should be inserted into the femoral head through the lateral wall of the proximal femur, which has the function of supporting the head–neck fragment and resisting its excessive sliding [2–4]. The lateral wall first plays a mechanical role through the anterior wall. The head–neck fragment should be supported by the anterior medial cortex as the head–neck fragment slides outward [5, 6].

The proximal cortex of the shaft refers to the cortex proximal to the trochanteric level of the femoral shaft. The remnant axial cortical length (RACL) refers to the proximal circumference of the femur around the lateral wall opening channel of the implant into the head–neck fragment when angled 130° upward to the fracture line in trochanteric hip fractures. This can be further divided into the remnant anterior cortex (RAC), remnant lateral cortex (RLC), and remnant posterior cortex (RPC). In 2016, Sharma et al. [7] found that the circumference of the lateral wall is associated with lateral wall fractures, and that a smaller circumference of the lateral wall is associated with an increased risk of a lateral wall fracture. However, there is still a lack of detailed analyses on the morphological characteristics of the RACL, which should be fully considered in the selection of surgical implants.

This study aimed to compare the characteristics of the RACL in A1 fractures with those in A2 fractures through computed tomography (CT) measurements, and to determine whether the RAC can be used as a sustainable region for anteromedial cortical reduction. We hypothesized that fracture reduction within the RAC is sufficiently consistent and reliable to maintain the postoperative stable reconstruction of per/inter-trochanteric fractures, thus providing good mechanical support for fracture healing.

## Patients and methods

This retrospective study was approved by the hospital ethics committee. The medical records of patients with AO Foundation and Orthopaedic Trauma Association (AO/OTA) 31-A1 and A2 trochanteric hip fractures who were treated with proximal femur nail antirotation (PFNA) were reviewed retrospectively from January 2015 to January 2016.

The inclusion criteria were as follows: a fresh fracture (within one week) at the age of 60 years or over; independently walking before the fracture; and complete CT imaging data confirming an anterograde intertrochanteric fracture (AO/OTA classification 31-A1 and A2). The exclusion criteria consisted of incomplete CT imaging data, an AO/OTA classification of an 31-A3 intertrochanteric fracture, a pathological fracture, or a secondary fracture of the ipsilateral femur.

All of the information was recorded, and all of the evaluations were performed by the senior visiting orthopedic staff. In all, 80 patients were identified who satisfied the inclusion criteria. According to the AO/OTA (2007) classification, 21 patients had A1 fractures and 59 patients had A2 fractures (see Table 1 for baseline data).

**Table 1:** Baseline characteristics of patients with AO/OTA 31-A1 and A2 intertrochanteric fractures

Parameters	AO/OTA fracture type			*P* value
	**Total (*n* = 80)**	**A1 (*n* = 21)**	**A2 (*n* = 59)**	
Age (years)	80.7 ± 5.8	79.6 ± 4.8	81.8 ± 5.7	0.132
Female, number (%)	32 (60)	8 (38)	24 (41)	0.836
Right, number (%)	41 (51.3)	13 (61.9)	28 (47.5)	0.255
RACL^*^ (mm)	67.6 ± 18.1	88.8 ± 15.8	60.0 ± 11.9	0.006
RAC^†^ (mm)	33.5 ± 6.7	36.9 ± 7.4	32.3 ± 6.1	< 0.001
RPC^†^ (mm)	13.9 ± 10.6	27.3 ± 7.8	9.2 ± 6.6	< 0.001
RLC^†^ (mm)	29.9 ± 7.1	36.9 ± 6.7	27.4 ± 5.4	< 0.001
γ angle^†^ (°)	33.9 ± 17.6	16.2 ± 13.1	40.3 ± 14.5	< 0.001
LWT^†^ (mm)	23.8 ± 7.2	32.1 ± 6.4	20.8 ± 4.6	< 0.001

^*^P < 0.01, ^†^*P* < 0.001: Comparison between A1 and A2 fractures

*RACL* Remnant axial cortical length, *RAC* Remnant anterior cortex, *RPC* Remnant posterior cortex, *RLC*, Remnant lateral cortex, *LWT* Lateral wall thickness

### Simulated reduction of fracture

**Fig. 1:**
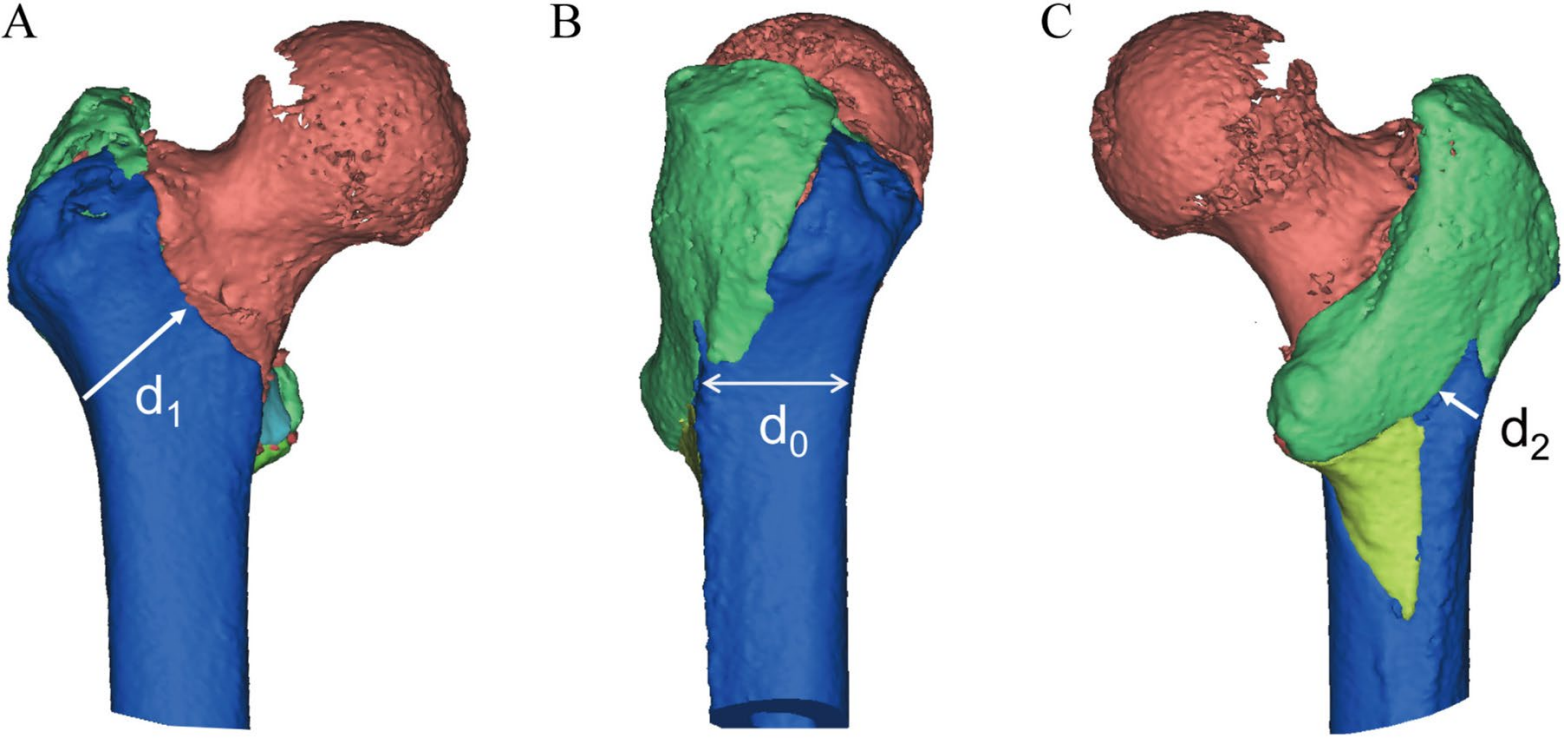
Illustration of 3D model of a pertrochanteric fracture after reconstruction. Each segment was reduced to normal anatomical positions; **A** anterior, **B** lateral, and **C** posterior views. d1: RAC, d0: RLC, and d2: RPC. Red: femoral head and neck, blue: femoral shaft, and green: posterior fragment. RAC: remnant anterior cortex, RLC: remnant lateral cortex, RPC: remnant posterior cortex

**Fig. 2:**
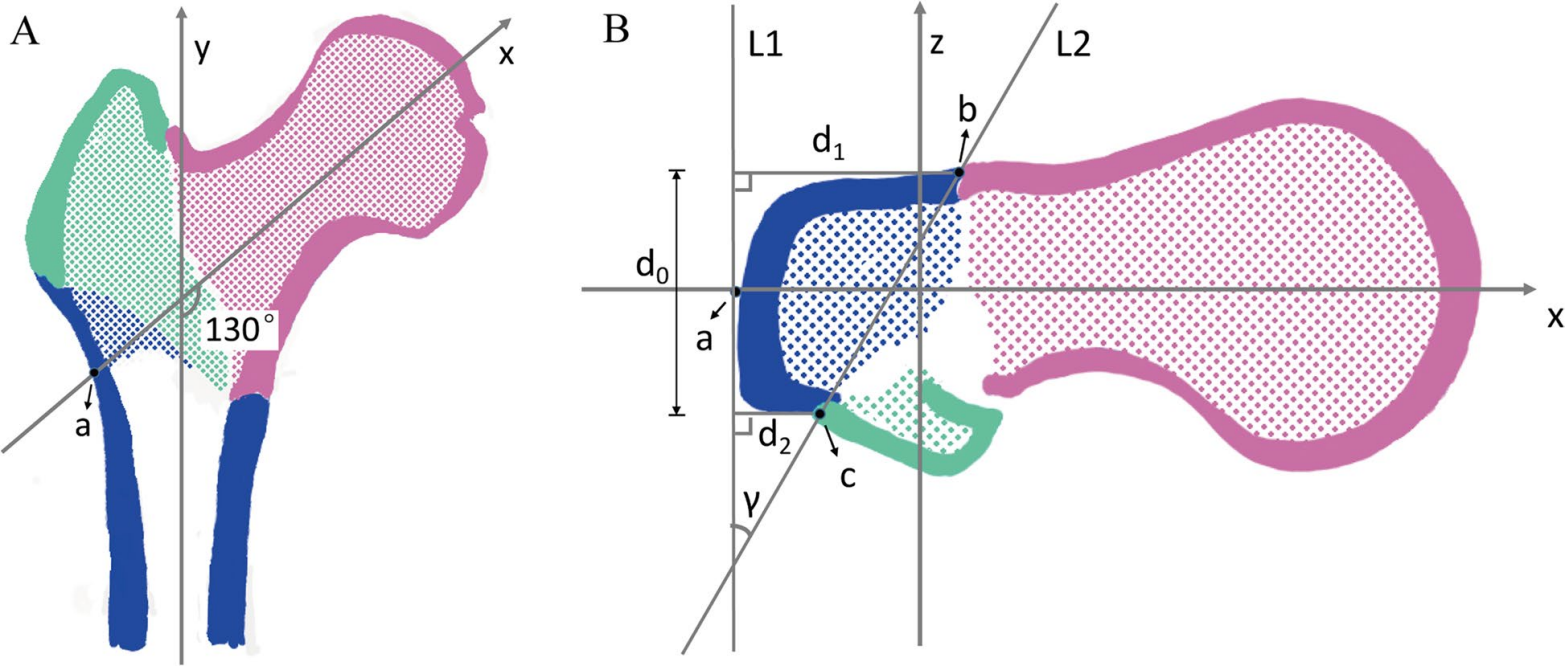
Illustration showing coronal (**A**) and axial (**B**) CT images of a patient with AO/OTA 31 A2 fracture. The central axis of the femoral head and neck was set as the x-axis, the central axis of the femoral shaft was set as the y-axis, the z-axis was perpendicular to the x-axis and y-axis, and the common plane of the x-axis and y-axis was set as the coronal plane. The axial CT plane was angled 130° upward to the femoral head and neck. The RACL of the proximal femur was measured as the length of the remnant lateral cortex on the central axis of the head and neck (blue line). Line 1 (L1) was a line tangential to the lateral wall of the proximal femoral fracture. Point (a) was the intersection of the x-axis and the lateral edge of the cortex, point (b) was a remnant anterior cortical extremity, and point (c) was a remnant posterior cortical extremity. Line 2 (L2) was a straight line passing through points (b) and (c). The γ angle was the angle between L1 and L2. The RACL was further divided into the RAC (d1), the RLC (d0), and the RPC (d2). Red: femoral head and neck, blue: femoral shaft, and green: posterior fragment. AO/OTA, AO Foundation and Orthopaedic Trauma Association, RAC: remnant anterior cortex, RACL: remnant axial cortical length, RLC: remnant lateral cortex, RPC: remnant posterior cortex

The CT data of the patients were imported into Mimics 17.0 (Materialise, Leuven, Belgium) in the Digital Imaging and Communications in Medicine (DICOM) format to reconstruct the three-dimensional structure of each fracture. By moving and rotating the fragment, the simulated reduction to the normal anatomic state was achieved (Fig. 1). The selection of the measurement plane was as follows: the central axis of the femoral shaft was set as the y-axis, the central axis of the femoral head and neck was set as the x-axis, with an angle of 130°, and the common plane of the x-axis and y-axis was set as the coronal plane; and the z-axis was perpendicular to the coronal plane (Fig. 2).

The auxiliary lines were set to mark the femoral neck as follows: line L1 was tangential to the lateral wall of the proximal femoral fracture; point (a) was the intersection of the x-axis and the lateral edge of the cortex, which was set as the drilling site of the screw blade or lag screw; point (b) was a remnant anterior cortical extremity; and point (c) was a remnant posterior cortical extremity (Fig. 2).

The following parameters were measured. The RACL was defined as the length of the RLC angled 130° upward on the x-axis. The length of the RAC (d1) was defined as the distance between line L1 and point (b). The length of the RPC (d2) was defined as the distance between line L1 and point (c). The length of the RLC (d0) was defined as the distance between the RAC and the RPC. The γ angle between the anterior and posterior cortex was measured by finding the angle between line L1 and a line joining points (b) and (c). The lateral wall thickness (LWT) was defined as the distance in mm from a reference point 3 cm below the innominate tubercle of the greater trochanter angled 135° upward to the fracture line [8] (the mean value of the sum of the RAC and RPC, Fig. 2).

### Statistical analysis

All of the statistical calculations were performed using SPSS version 19.0 (SPSS Inc. Chicago, IL, USA). Student’s *t* tests and one-way analyses of variance (ANOVAs) were used for continuous variables, and χ^2^ tests or Fisher’s exact tests were used for categorical variables. Linear regression was used to determine the correlation between variables. Statistical significance was defined as *P* < 0.05.

## Results

This study compared the RACL between type A1 (21 cases) and type A2 (59 cases) pertrochanteric fractures (total 80 cases). The mean RACL in patients with A1 fractures was 88.8 ± 15.8 mm, while that in patients with A2 fractures 60.0 ± 11.9 mm. Although there were statistical differences between these two fracture types in the three components of RACL, the most significant difference was in the RPC, which was 27.3 ± 7.8 mm in A1 fractures and 9.2 ± 6.6 mm in A2 fractures (*P* < 0.001, Table 1).

### Lengths of the remnant anterior, lateral and posterior cortices

**Fig. 3:**
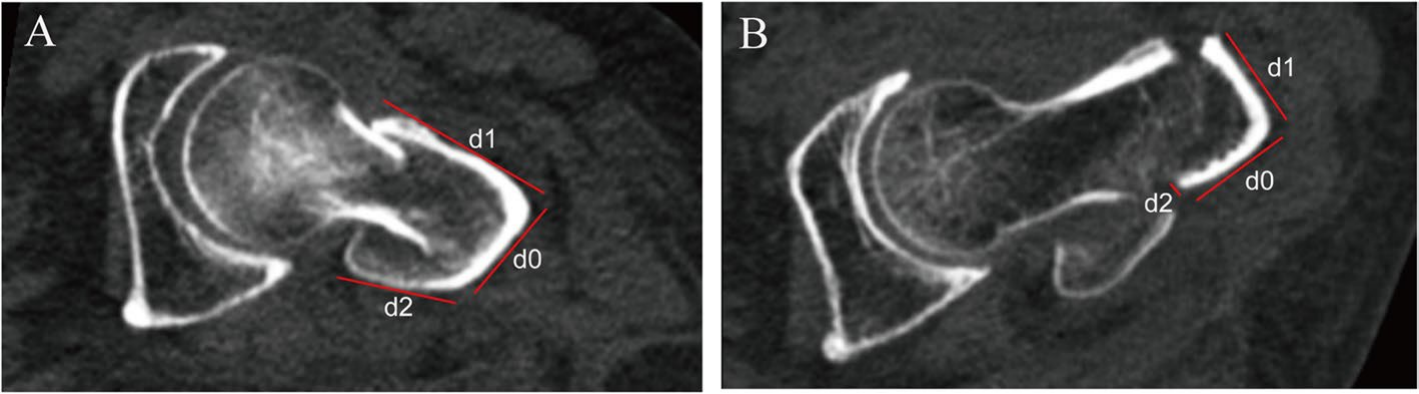
Representative CT images showing the RAC (d1), RLC (d0) and RPC (d2) of the RACL in patients with an A1 fracture (**A**) and in patients with an A2 fracture (**B**). The RAC, RPC, and RPC (red line) in A1 fractures were significantly longer than those in A2 fractures. Patients with A2 fractures involved the posterior wall fragment. RAC: remnant anterior cortex, RACL: remnant axial cortical length, RLC: remnant lateral cortex, RPC: remnant posterior cortex

The data of the 80 patients with AO/OTA 31-A1 and A2 trochanteric hip fractures are summarized in Table 1. In all patients, the average length of the RAC was 33.5 ± 6.7 mm, which was much larger than that of the RPC (13.9 ± 10.5 mm; *P* < 0.01). The coefficient of variation of the RAC was only 20.0%, which was the smallest part, while that of the RPC was 75.5%, which was the largest part. Out of 59 patients with A2 fractures, the length of the RPC was < 5 mm in 18 patients, and their true posterior cortices were entirely broken and displaced; that is, the RPC was 0 mm (31%, 18/59 cases; Table 1). The representative morphology of the RACL is shown in Fig. 3. There were significant differences in the RACL, RAC, RLC, RPC, γ angle, and LWT among the five subtypes (*P* < 0.001, Table 2).

**Table 2:** Parameters among subtypes of AO/OTA 31-A1 and A2 intertrochanteric fractures

Parameters	A1.1 (*n* = 5)	A1.2 (*n* = 16)	A2.1 (*n* = 38)	A2.2 (*n* = 15)	A2.3 (*n* = 6)	*P* value
RACL^*^ (mm)	97.4 ± 12.9	86.0 ± 15.9	62.8 ± 12.0	57.8 ± 9.3	47.9 ± 8.1	< 0.001
RAC^*^ (mm)	39.1 ± 6.9	36.2 ± 7.5	34.0 ± 5.0	30.9 ± 6.6	24.8 ± 4.8	< 0.001
RPC^*^ (mm)	29.9 ± 4.7	26.5 ± 8.4	9.8 ± 7.5	8.8 ± 4.4	6.3 ± 3.5	< 0.001
RLC^*^ (mm)	37.7 ± 5.7	36.7 ± 7.1	28.2 ± 5.7	26.5 ± 4.7	24.1 ± 3.3	< 0.001
γ angle^*^ (mm)	15.0 ± 13.0	16.5 ± 13.4	40.9 ± 15.7	39.2 ± 12.9	38.8 ± 10.5	< 0.001
LWT^*^ (mm)	34.5 ± 4.3	31.4 ± 6.9	21.9 ± 4.5	19.9 ± 2.9	15.6 ± 3.0	< 0.001

^*^P < 0.001: Comparisons of RACL, RAC, RLC, RPC, γ angle, and LWT among the five subtypes

*RACL* Remnant axial cortical length, *RAC* Remnant anterior cortex, *RPC* Remnant posterior cortex, *RLC* Remnant lateral cortex, *LWT* Lateral wall thickness

There was a significant difference in the LWT between A1 and A2 fractures (*P* < 0.001). Eighteen patients with A2 fractures had a partial ruptured length of the RLC on the coronal plane; all of them involved a posterior wall fragment but did not exceed the midline of the RLC. The posterior wall sometimes included the greater trochanter, the intertrochanteric ridge, the lesser trochanter, or various combinations of these characteristics. A total of 55 patients with A2 fractures had a posterior fragment (93.2%, 55/59 patients), while 21 (35.6%) patients had a banana-shaped fragment composed of the greater trochanter, an intertrochanteric ridge, and the lesser trochanter.

### The γ angle between the remnant anterior and posterior cortices

The difference between the RAC and the RPC was reflected by measuring the γ angle between the sagittal line and the line joining the RAC and the RPC extremities in the sagittal axis. The γ angle in A1 fractures was 16.2 ± 13.1°, which was significantly smaller than the γ angle of 40.3 ± 14.5° in A2 fractures (*P* < 0.001; Table 1).

**Fig. 4:**
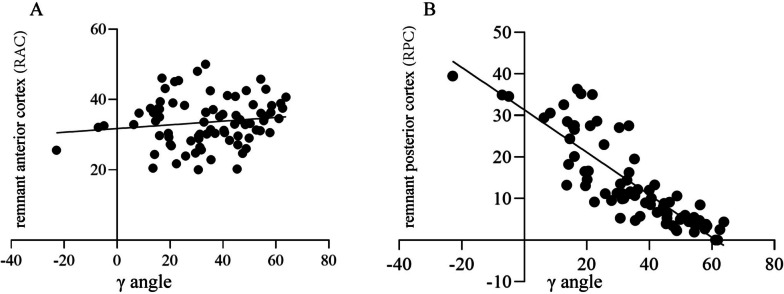
Correlation analyses between the RAC/RPC and the γ angle in trochanteric fractures. Linear regression analyses showed that there was no correlation between the RAC and the γ angle (**A**), and that there was a linear negative correlation between the γ angle and the RPC (**B**). RAC: remnant anterior cortex, RPC: remnant posterior cortex

Linear regression analysis showed that the γ angle was significantly correlated with the length of the RPC (*r* = -0.854, *P* < 0.001), but not with the RAC (r = 0.138, *P* = 0.222; Fig. 4). Furthermore, the length of the RAC was stable.

## Discussion

The purpose of this study was to compare the characteristics of the RACL in A1 fractures with those in A2 fractures and to determine the feasibility of anteromedial cortical reduction using the RAC. It was found that the RAC, RLC, and RPC in A1 fractures were significantly longer than those in A2 fractures. In addition, the coefficient of variation of the RAC was smaller than that of the RPC. The γ angle in A1 fractures was significantly smaller than that in A2 fractures. Therefore, fracture reduction through a RAC buttress may enhance the postoperative stability of per/intertrochanteric fracture reconstruction, thus mechanically supporting fracture healing.

The proximal femoral shaft is the basis of reduction and fixation of trochanteric hip fractures, providing a natural cortical buttress for the head–neck fragment and fixation. Several studies have been performed to investigate the risk factors associated with internal fixation failure or reoperation after trochanteric hip fracture. In 2004, Gotfried [3] emphasized that in unstable pertrochanteric hip fractures, the integrity of the lateral wall should be considered the most important prognostic factor in determining the internal fixation device used for fracture stabilization. In 2007, Palm et al. [9] reported that a postoperative fracture of the lateral femoral wall was the main predictor of the need for reoperation after trochanteric femur fracture. In 2013, Hsu et al. [8] first proposed that if the lateral wall thickness was < 20.5 mm, the lateral wall was unable to provide support, vulnerable to a lateral wall fracture post-operation and was at a significantly increased risk of fixation failure. However, the measurement of emergency X-rays have some shortcomings, such as unclear photography, overlapping shadows, difficulties in accurately identifying anterior and posterior cortices, and limb rotations.

CT has been used in trochanteric fractures for assessing the reliability of classification systems [10, 11]. In 2016, Sharma et al. [7] reported that 51 patients with AO/OTA 31-A2 pertrochanteric fractures were evaluated using a pre-operative CT scan, and found that AO/OTA 31-A2 pertrochanteric fractures with a lateral wall height of > 1.68 cm and an anterior component of > 2.10 cm in circumference are not likely to sustain a lateral wall fracture when treated with a dynamic hip screw (DHS). However, due to no reduction of fragments, there are some shortcomings, such as a lack of accurate calibration, greater subjectivity, and inaccurate data. Our study overcame such shortcomings; the fragments were returned to the normal position through the simulated reduction of each fragment, the advantage of which was the ability to obtain more accurate and objective data that may better reflect the true state of the fracture.

The anterior cortex is characterized by a large residual length/thickness and hard texture, providing a natural cortical buttress for the head–neck fragment and fixation, which can be considered as the most stable part of the circumference of the RACL [12, 13]. When an iatrogenic fracture occurs at the insertion of the lateral wall into the nail, the anterior wall is also concurrently damaged. In such cases, the head–neck fragment will lose the support of the anterior and lateral cortices, which will greatly increase the risk of a postoperative-fatigue fracture. This study showed that for both A1 and A2 fractures, the length of the RAC angled at 130° upward was approximately 3 cm, meaning it runs approximately along the intertrochanteric line of the anterior capsular attachment, and that the RAC was the most reliable support structure of the RACL. Our results are consistent with those of previous studies [14, 15].

The lateral cortex itself is relatively thin and is used for the drilling site of head and neck implants, which contribute to the lateral cortex being subject to a high incidence of iatrogenic fractures. A fracture of the lateral cortex changes an original A1/A2 fracture pattern into an unstable A3 fracture [16]. It has been reported that a lateral wall fracture takes place in 21% of cases following intramedullary nail and DHS [17]. In 2023, Li et al. [18] found that rupture of the remnant lateral wall is a high risk of mechanical complications in intertrochanteric fractures, and that a residual lateral wall width of 18.55 mm is a reliable predictor of postoperative rupture of the entry portal. The main determinants of an iatrogenic fracture of the lateral cortex are its fracture pattern, including its fracture line trend, and also the length, height, and area of the lateral wall.

The length of the RLC is mainly affected by the posterior fragments, while the height is mainly affected by the greater trochanter fragments [19]. In the study, 93% of A2 fractures were in the presence of a posterior medial fragment, while the length of the RLC was invariably affected by the posterior fragment (100%). The coronal fracture line of the posterior wall always ran inferiorly through the lesser trochanter or the posteromedial cortex. Lower coronal fracture lines were found to have more obvious damage to the length of the RLC. The posterior medial fracture line in A2 fractures was lower, and it reached the lesser trochanter and the lower part. However, the fracture line of the posterior wall on the lateral coronal plane rarely exceeded the midpoint of the lateral wall. Therefore, the anterolateral cortex in the proximal femur was basically intact.

In this study, posterior wall factures were common and their morphologies were complex, which had smaller average lengths and greater variations than those of the RAC. The γ angle between the anterior and posterior cortex represents the difference in length between the RAC and the RPC. When the angle is 0°, it indicates that the lengths of the anterior and posterior cortices are equal. Larger angles reflect greater differences between the RAC and the RPC. This study showed that the average angle in A2 fractures was 40.2°, which was approximately three times that in A1 fractures. Linear analysis showed that the γ angle was mainly affected by the lengths of the RPC, which further confirmed the stability of the RAC.

The lateral wall area (height × length) is mainly affected by posterior coronal fractures [20]. In the study, it was found that the posterior fragment rarely affected the anterior lateral wall. The total length of the anterior part of the RLC and the RAC was 4–5 cm. Hence, protecting and making good use of the anterolateral cortex may be a key for improving the stability of internal fixation for trochanteric fractures.

In 2013, Hsu et al. [8] found that the mean LWT in A1 fractures was 29.8 ± 6.63 mm, which was significantly thicker than the mean of 21.2 ± 6.43 mm in A2 fractures, and that LWT still significantly contributed to lateral wall fracture in A2 fractures. This study found that the LWT of A1 fractures was 32.13 ± 6.4 mm, while that of A2 fractures was 20.76 ± 4.6 mm. The reason for the difference between these two studies may be that the former is based on ordinary X-ray measurement; measuring LWT based on radiographs taken in the emergency department is difficult. Because the distal fragment is externally rotated, identifying the distal end of the innominate tubercle of the greater trochanter becomes challenging. The measurement of LWT will also vary significantly in the same fracture depending on the degree of rotation. In contrast, the measurements in this study were taken based on three-dimensional CT and fracture reduction. The measurement error of this method is small and can reflect the real shape of the fracture.

There were several limitations of this study. First, the relationship between different fracture morphologies and functional prognoses were not explored, and there were no follow-ups of the internal fixation effects of patients. Second, our process of fracture classification mainly depended on subjective assessments. Although fractures were observed under three-dimensional reconstructed images, the influences of subjective factors cannot be excluded for some intermediate fractures.

## Conclusions

In this study, we found that the RACL in A2 fractures was much smaller than that in A1 fractures, primarily due to the posterior coronal fragments. The RAC was relatively stable. Fracture reduction with an RAC buttress was reliable enough for the postoperative stable reconstruction of per/inter-trochanteric fractures, providing a good mechanical support for fracture healing.

## Data Availability

The datasets used and/or analysed during this study are available from the corresponding author upon reasonable request.

## Abbreviations

AO/OTA: AO Foundation and Orthopaedic Trauma Association
CT: Computed tomography
RACL: Remnant axial cortical length
RAC: Remnant anterior cortex
RPC: Remnant posterior cortex
RLC: Remnant lateral cortex
PFNA: Proximal femur nail antirotation
DICOM: Digital Imaging and Communications in Medicine
LWT: Lateral wall thickness
